# Stability of Refrigerated Medications at Room Temperature: Implications for Transport, Delivery, and Patient Safety

**DOI:** 10.7759/cureus.93213

**Published:** 2025-09-25

**Authors:** Faisal A Aleidi, Samar Alomair, Huda Alharbi, Norah AlSalamah, Lamya AlEnazi

**Affiliations:** 1 Pharmacy Service Administration, King Fahad Medical City, Riyadh, SAU

**Keywords:** heat, refrigerator, room temperature, stability, storage

## Abstract

Refrigeration is essential for maintaining the stability and efficacy of many pharmaceuticals, including vaccines, biologics, and certain antibiotics. However, unintentional exposure of refrigerated medications to room temperature may occur during transport delays, power outages, or handling errors, raising concerns about compromised potency and patient safety. Despite frequent real-world cold chain breaches, evidence-based guidance on acceptable room temperature excursions remains limited.

This study aimed to review and summarize available data regarding the stability of refrigerated medications when exposed to room temperature, providing a practical reference tool for healthcare providers. All U.S. Food and Drug Administration (FDA)-approved unopened refrigerated pharmaceutical and biological products up to June 30, 2024, were screened from our institutional formulary. Stability data were collected from drug SmPCs, package inserts, regulatory agency databases (FDA, European Medicines Agency (EMA), Saudi Food and Drug Authority (SFDA)), published literature, and tertiary drug information resources (Lexicomp, Micromedex, UpToDate). Where data were lacking, manufacturers were contacted directly. Medications were categorized based on documented stability at room temperature (20°C-25°C), and a reference table was developed summarizing acceptable excursion durations.

A total of 150 refrigerated medications were identified with available data supporting room temperature stability. Out of them, 22.8% of the products remained stable for at least 24 hours, while several demonstrated stabilities for extended durations. Notable variability was observed across brands containing the same active substance, such as recombinant human growth hormone products.

This review provides an updated, comprehensive resource summarizing the stability of refrigerated medications at room temperature. The findings underscore the clinical and economic importance of minimizing unnecessary wastage following cold chain excursions. Brand-specific data must be prioritized, as stability profiles differ even among products with the same active ingredient. Broader transparency from pharmaceutical manufacturers regarding temperature excursion studies is urgently needed to support evidence-based decision-making in pharmacy practice, patient safety, and cost containment.

## Introduction and background

Refrigeration is a crucial component in ensuring the efficacy and safety of various medications, particularly biologics, vaccines, and certain injectable drugs. However, situations arise in which refrigerated medications may be exposed to room temperature for periods due to transport delays, power outages or unintentional exposure to heat [1,2]. In some cases, medications may also be forgotten at room temperature for extended periods, compromising their stability. These unintended deviations from recommended storage conditions can potentially affect the potency, safety, and overall quality of the medication, leading to concerns regarding their continued effectiveness and safety for patient use [3].

Current stability data for medications primarily emphasize storage recommendations under controlled conditions. However, comprehensive research on the stability of refrigerated medications when exposed to room temperature remains limited despite the increasing frequency of such scenarios in real-world settings. Numerous medications - such as vaccines, biologics (e.g., insulin and monoclonal antibodies) and certain antibiotics - require refrigeration to maintain their stability and efficacy [4,5]. Nevertheless, clear guidelines on the permissible duration of storage at room temperature are lacking. While drug package inserts often caution that efficacy may be compromised if storage conditions are not maintained, they rarely provide specific data regarding the exact time frame within which these medications remain stable outside of refrigeration.

There have been documented instances where refrigerated medications were exposed to room temperature unintentionally. For example, the U.S. Centers for Disease Control and Prevention (CDC) and the World Health Organization (WHO) have issued alerts about temperature excursions affecting the efficacy of vaccines. These occurrences highlight the importance of having reliable data on how various medications behave when exposed to higher temperatures [6].

In Cohen V et al. study, the researchers compiled data on the safe room-temperature storage duration of refrigerated medications available in hospital pharmacies. The researchers reviewed prescribing information and conducted manufacturer surveys to gather details on 79 medications labeled for storage at 2°C-8°C. The U.S. Pharmacopeia's definition of room temperature (20°C-25°C) [3,7] was used in this study. These resources often provide general guidelines but lack specific details on what happens when refrigerated medications are exposed to room temperature for different periods [7]. Suárez-Casillas et al. (2025) in their review “Stability of Thermolabile Drugs at Room Temperature” provide valuable and updated information; however, the study only includes medications available in their hospital formulary rather than the full scope of Food and Drug Administration (FDA)- or European Medicines Agency (EMA)-approved agents and continuous updates and broader inclusion of international drug lists are needed to ensure comprehensive coverage and applicability across different healthcare settings [8].

Orth et al. (2022) conducted a review assessing the stability of refrigerated medications during room temperature excursions. The study compiled data on acceptable storage durations based on prescribing information and manufacturer input. While the findings are valuable for pharmacy practice, the focus was limited to medications handled within clinical or pharmacy settings. A key limitation is the exclusion of medications stored or used by patients at home. In real-world scenarios, cold chain breaches often occur during transport or due to power outages. These situations are not addressed in the review. Further research is needed to evaluate medication stability in home-use conditions [9].

Medication stability is a common concern, especially during emergency situations or power outages. The lack of clear and standardized data presents a significant challenge for patients who rely on medications that require specific storage temperatures. In such cases, providers often consult drug information resources such Lexicomp, Micromedex, and the drug leaflets for guidance on medication storage and stability.

This review aimed to expand on earlier research by determining how long refrigerated medications can safely remain at room temperature without losing their effectiveness or stability. With the rapid increase in the development of thermolabile drugs in recent years, there is a growing demand for accurate storage information. Often, details about room temperature stability are missing from key resources like the Summary of Product Characteristics (SmPC), making it necessary to seek additional information. To address this, the study gathered and organized updated data from SmPCs, scientific literature, and pharmaceutical manufacturers to offer clear and practical guidance on the safe handling of these temperature-sensitive medications.

## Review

Method

All U.S. FDA-approved refrigerated pharmaceutical and biological products available in our tertiary hospital formulary up to June 30, 2024, were screened, with the review limited to unopened vials and products in our institutional formulary. Controlled room temperature was defined as 20°C-25°C according to the United States Pharmacopeia (USP) guidelines, and reported data on excursions, re-refrigeration, and cumulative time were extracted primarily from SmPCs. Additional sources included package inserts, regulatory agency databases (FDA, EMA, Saudi Food and Drug Authority (SFDA)), published literature, and tertiary drug information resources (Lexicomp, Micromedex, UpToDate). Where data were lacking, manufacturers were contacted directly via telephone or email to obtain unpublished stability information. Medications were then categorized as Stability in Refrigerator and Stability in Room Temperature (20°C-25°C) based on available data regarding potency retention and degradation. A comprehensive summary table was created to document the acceptable duration of room temperature excursions. Furthermore, drug monographs from Lexicomp, UpToDate, and IBM Micromedex were reviewed for all refrigerated pharmaceutical and biological products. When stability data were not available in these resources, direct manufacturer contact was made to supplement the findings.

Results

A total of 150 refrigerated items were identified with documented data supporting storage at room temperature. Evidence was mainly derived from the SmPCs and available published stability studies [7-11]. The detailed list of these items, along with the reported duration of stability and corresponding references, is presented in Table 1.

**Table 1:** Duration of Room Temperature Stability for Refrigerated Drugs with Supporting References Data adapted from references [7,8,10–12]. SmPC: Summary of Product Characteristics.

medication	Dosage Form	Brand Name	Stability in Refrigerator	Stability in Room Temperature	Reference
Acetylcholine	Intraocular Solution vial	Miochol®-E	4°C	25°C	SmPC
Adalimumb	Pen-injector	Humira®	2°C to 8°C	14 days	SmPC
Aflibercept	Solution intravitreal	Pavblu™	2°C to 8ºC	3 days	SmPC
Aflibercept	Solution intravitreal	Eylea™	2°C to 8ºC	24 h	SmPC
Aflibercept	Solution intravitreal	Eylea® HD	2°C to 8ºC	25 h	SmPC
Anakinra	Prefilled syringe	Kineretä	2° to 8°C	3 days	SmPC
Anidulafungin	Solution vial	Eraxis®	2°C to 8°C	96 hours up to 25°C are permitted, and the vial can be returned to storage at 2°C – 8°C	SmPC
Anifrolumab-fnia	Solution vial	Saphnelo	2°C to 8°C	The product should be not be exposed to temperatures of 8 to 25°C for longer than 21 days between 8 to 25°C, and the product should not be exposed to temperatures between 25 to 40°C for any longer than 4 hours.	SmPC
Balanced Salt Solution	Solution, Irrigation drops	(BSS®)	2°C to 8°C	25°C	SmPC
Belimumab	Autoinjector or prefilled syringe	Benlysta®	2°C to 8°C	Up to 30°C for 12 hours	SmPC
Benralizumab	Solution auto-injector	Fasenra®	2°C to 8°C	25°C up to 14 days	SmPC
Benzylpenicilloyl Polylysine	Solution injection vial	Pre-pen®	2°C to 8°C	25°C up to 24 hours	SmPC
Beractant	Suspension intratracheal	Survanta®	2°C to 8°C	Unopened, unused vials of Survanta that have been warmed to room temperature may be returned to the refrigerator within 24 hours of warming and stored for future use. Survanta should not be removed from the refrigerator for more than 24 hours.	SmPC
Bevacizumab [8]	Solution vial	Avastin®	2°C to 8°C	5 days at 15°C and 9 h at less than 30°C	
Blinatumomab	Solution vial	Blincyto®	2°C to 8°C	23°C to 27°C for 8 hours	SmPC
Botulism Antitoxin Heptavalent	Solution vial	BAT®	Freezer (-15°C) or below 5°C	Once thawed, Botulism Antitoxin Heptavalent (A, B, C, D, E, F, G) – (Equine) may be stored at 2-8°C (36-48°F) for a maximum of 36 months or until 48 months from the date of manufacture, whichever comes first	SmPC
Brentuximab Vedotin [8]	Solution vial	Adcetris®	2°C to 8°C	14 days	
Brinzolamide and Brimonidine	Ophthalmic drop	Simbrinza™	2°C - 8°C	25°C	SmPC
Burosumab-twza	Solution vial	Crysvita®	2°C to 8°C	48 h	SmPC
Calcitonin	Nasal Spray	Miacalcin®	2°C to 8°C	35 days	SmPC
Calcitonin	Solution injection vial	Miacalcin®	2°C to 8°C	14 daYS	SmPC
Calfactant	Suspension Intratracheal	Infrasurf®	2°C to 8°C	Up to 24 hours. Vials that have reached room temperature within 24 hours can be refrigerated for future use; however, do not re-refrigerate INFASURF more than once.	SmPC
Certolizumab Pegol	Prefilled Syringe	Cimzia	2 to 8 °C	7 days	SmPC
Cetrorelix Acetate	Injection vial	Cetrotide®	2 °C to 8 °C	3 months	SmPC
Cetuximab	Injection vial	Erbitux®	2 °C to 8 °C	8 hours	SmPC
DAPTOmycin (reconstituted) [7]	Solution injection vial	Cubicin®	2°C to 8°C	12 months	
Daratumumab and Hyaluronidase	Solution injection vial	Darzalex Faspro®	2°C to 8°C	24 h. Protect from light	SmPC
Darbepoetin Alfa	Prefilled syringe	Aranesp®	2°C to 8°C	7 days	SmPC
Denosumab	Prefilled syringe	Prolia®	2°C to 8°C	25°C up to 30 days	SmPC
Denosumab	Prefilled syringe	Xgeva®	2°C to 8°C	25°C up to 30 days	SmPC
Digoxin Immune Fab	injection vial	Digibind®	2°C to 8°C	Up to 30 days	SmPC
Dornase Alfa	Solution, Inhalation	Pulmozyme®	2°C to 8°C	Do not use Pulmozyme if the ampules have been exposed to room temperature at 72°F to 82°F (22°C to 28°C) for more than a total of 60 hours or if the solution has turned cloudy or discolored.	SmPC
Dupilumab	Prefilled syringe	Dupixent®	2°C to 8°C	25°C up to 14 days	SmPC
Durvalumab	Solution injection vial	Imfinzi®	2°C to 8°C	8 hours	SmPC
Eftrenonacog Alfa (Factor IX (Recombinant (FC Fusion Protein))	Solution injection vial	Alprolix	2°C to 8°C	25°C up to 6 months	SmPC
Emicizumab	Solution injection vial	Hemlibra®	2°C to 8°C	Below 30°C up to 7 days	SmPC
Etanercept	Prefilled autoinjector	Enbrel®	2°C to 8°C	25°C for up to 30 days	SmPC
Etoposide	Injection vial	VePesid®	2° to 8°C	24 months	SmPC
Etoposide	Solution injection vial	Etoposide® Injection	2° to 8°C	A shelf life of 24 months from the date of manufacture is recommended when stored below 25°C	SmPC
Evolocumab	Prefilled syringe	Repatha	2°C to 8°C	20°C to 25°C up to 30 days	SmPC
Fibrinogen Concentrate (Human)	Injection vial	Haemocomplettan®	2°C to 8°C	8 hours	SmPC
Fibrinogen Concentrate (Human)	Injection vial	Fibryga	NA	25°C	SmPC
Fibrinogen Concentrate (Human)	Injection vial	Riastap®	NA	25°C	SmPC
Filgrastim	Injection vial	Neupogen®	2°C to 8°C	Up to 7 days	SmPC
Filgrastim	Injection vial	Nypozitm	2°C to 8°C	Up to 24 hours	SmPC
Filgrastim	Injection vial	Nivestymtm	2°C to 8°C	Up to 24 hours	SmPC
Fulvestrant	Solution injection vial	Faslodex®	2°C to 8°C	28 days	SmPC
Fusidic Acid (Ophthalmic)	Suspension ophthalmic	Fucithalmic®	2°C to 8°C	25°C	SmPC
Galcanezumab	Solution auto-injector	Emgality®	2°C to 8°C	30°C up to 7 days	SmPC
Guselkumab [8]	Prefilled Syringe	Tremfya®	2°C to 8°C	24 h	
Hepatitis A Vaccine	Solution injection vial	Havrix®	2°C to 8°C	72 hours	SmPC
Hepatitis A vaccine	Solution injection vial	Vaqta	2°C to 8°C	72 hours	SmPC
Hepatitis B Vaccine	Solution injection vial	Engerix-B®	2°C to 8°C	25°C up to 72 hr	SmPC
Hepatitis B Vaccine	Solution injection vial	Recombivax HB	2°C to 8°C	25°C up to 72 hr	SmPC
Hyaluronidase [10]	Injection vial	Hylenex®	2°C to 8°C	12 months	
InFLIXimab	Injection vial	Remicade®	2°C to 8°C	Remicade® can also be stored in the original carton outside of refrigerated storage up to a maximum of 30°C for a single period of up to 6 months; but not exceeding the refrigerated expiration date printed on the carton.	SmPC
InFLIXimab	Prefilled syringe	Remisima SC	2°C to 8°C	Maximum of 25°C for a period of up to 28 days	SmPC
InFLIXimab	Prefilled syringe	Zymfentra	2°C to 8°C	25°C up to 14 days	SmPC
Inotuzumab Ozogamicin [8]	Solution injection vial	Besponsa®	2°C to 8°C	1 year	
Insulin Aspart	PenFill cartridges	NovoLog®	2°C to 8°C	Below 30°C up to 28 days	SmPC
Insulin Aspart	Flex touch	NovoLog®	2°C to 8°C	Below 30°C up to 28 days	SmPC
Insulin Aspart	Vial	NovoLog®	2°C to 8°C	Below 30°C up to 28 days	SmPC
Insulin Aspart	FlexPen	NovoLog®	2°C to 8°C	Below 30°C up to 28 days	SmPC
Insulin Aspart Protamine and Insulin Aspart	Vials	NovoLog® Mix 70/30	2°C to 8°C	Below 30°C up to 28 days	SmPC
Insulin Aspart Protamine and Insulin Aspart	FlexPen	NovoLog® Mix 70/30	2°C to 8°C	Below 30°C up to 14 days	SmPC
Insulin Degludec	Solution pen-injector	Tresiba U 100 FlexTouch	2°C to 8°C	Below 30°C up to 56 days (8 weeks)	SmPC
Insulin Degludec and Insulin Aspart	70/30 100 units/mL (U-100) in: 3 mL	Ryzodeg® FlexTouch	2°C to 8°C	Below 30°C up to 28 days	SmPC
Insulin Detemir	Solution pen-injector	Levemir® FlexPen	2°C to 8°C	Below 30°C up to 42 days	SmPC
Insulin Glargine	Prefilled syringes	Lantus® prefilled pen	2°C to 8°C	Below 30°C up to 28 days	SmPC
Insulin Glargine	Prefilled syringes	Toujeo®	2°C to 8°C	Below 30°C up to 56 days	SmPC
Insulin Glargine	Multiple-dose vial	Lantus®	2°C to 8°C	Below 30°C up to 28 days	SmPC
Insulin Glargine &Lixisenatide	Pen-injector	Soliqua™ 100/33	2°C to 8°C	After first use, store your Soliqua 100/33 pen at room temperature no higher than 77°F (25°C) for 28 days.	SmPC
Insulin Human Isophane	KwikPen prefilled pen	Humulin®N	2°C to 8°C	Below 30°C up to 14 days	SmPC
Insulin Human Isophane	Multiple-dose vial	Humulin®N	2°C to 8°C	Below 30°C up to 31 days	SmPC
Insulin Human Isophane	Multiple-dose vial	Novolin® N	2°C to 8°C	Up to 25°C for 42 days	SmPC
Insulin Human Isophane	FlexPen prefilled pen	Novolin® N prefilled pen	2°C to 8°C	Up to 25°C for 28 days	SmPC
Cisatracurium (presevative-free)	Solution injection vial	Nimbex®	2°C to 8°C	Within 21 days	SmPC
Insulin NPH and insulin regular	multiple-dose vial	Humulin® 70/30	2°C to 8°C	Below 30°C up to 31 days	SmPC
Insulin NPH and insulin regular	KwikPen prefilled pen	Humulin® 70/30 KwikPen	2°C to 8°C	Below 30°C up to 10 days	SmPC
Insulin NPH and insulin regular	Multiple-dose vial	Novolin® 70/30	2°C to 8°C	Up to 25°C up to 42 days	SmPC
Insulin NPH and insulin regular	Prefilled pen	Novolin® 70/30	2°C to 8°C	Up to 25°C up to 28 days	SmPC
Insulin regular	Multiple-dose vial	Humulin®R	2°C to 8°C	Below 30°C up to 31 days	SmPC
Insulin regular	FlexPen prefilled pen	Novolin®R	2°C to 8°C	Below 30°C Up to 28 days	SmPC
Insulin regular	Multiple-dose vial	Novolin®R	2°C to 8°C	Below 25°C Up to 42 days	SmPC
Interferon Beta-1a	Vial	Avonex®	2°C to 8°C	25°C up to 30 days	SmPC
Interferon Beta-1a	Prefilled syringe	Avonex®	2°C to 8°C	25°C up to 7 days	SmPC
Interferon Beta-1a	Autoinjector	Rebif®	2°C to 8°C	25°C up to 30 days	SmPC
Interferon Gamma-1b	100 mcg [2 million int. Units] (0.5 ml)	Actimmune®	2°C to 8°C	Up to 12 hours	SmPC
Ipilimumab [11]	Solution injection vial	Yervoy®	2°C to 8°C	28 days	
Lanreotide	Solution subcutaneous injection	Somatuline® Depot	2°C to 8°C	Product left in its sealed pouch at room temperature (not to exceed 104°F or 40°C) for up to 72 hours may be returned to the refrigerator for continued storage and use at a later time	SmPC
Liraglutide	Pen-injector, subcutaneous	Saxenda®	2°C to 8°C	After initial use of the Saxenda pen, the pen can be stored for 30 days at controlled room temperature	SmPC
Liraglutide	Pen-injector, subcutaneous	Victosa®	2°C to 8°C	After first use of the Victosa pen, the pen can be stored for 30 days at controlled room temperature (59°F to 86°F; 15°C to 30°C) or in a refrigerator (36°F to 46°F; 2°C to 8°C).	SmPC
Lutropin Alfa	Injection vial	Luveris®	2°C to 25°C	2°C to 25°C	SmPC
Meningococcal Group B Vaccine [7]	Prefilled syringe	Bexsero®	2°C to 8°C	48 h	
Methylergonovine maleate [12]	Solution injection vial	Methergine®	2°C to 8°C	12 months	
Moxifloxacin (ophthalmic)	Solution ophthalmic drop	Vigamox®	2°C to 8°C	Not exceed 25°C	SmPC
Natalizumab	Prefilled injection	Tysabri®	2°C to 8°C	Up to 25°C: 24 hours	SmPC
Neomycin, Polymyxin B, and Dexamethasone	Ophthalmic suspension	Maxitrol®	8°C	27°C	SmPC
Nivolumab	Solution injection vial	Opdivo®	2 to 8 °C	48 h	SmPC
Ocrelizumab	Solution injection vial	Ocrevus®	2°C to 8°C	12 h	SmPC
Octreotide	Solution injection vial	Sandostatin®	2°C to 8°C	20°C to 30°C up to 14 days	SmPC
Ofatumumab	Solution auto-injector	Kesimpta®	2°C to 8°C	7 days at room temperature, not to exceed 30°C (86°F). Write the date removed from the refrigerator in the space provided on the carton labeling. If stored below 30°C (86°F), unused Kesimpta may be returned to the refrigerator and must be used within the next 7 days or discarded after 7 days.	SmPC
Olopatadine (Ophthalmic)	Solution, ophthalmic drop: 0.2%	Pataday®	4°C	Up to 25°C	SmPC
Omalizumab	Injection vial	Xolair®	2°C to 8°C	2 days	SmPC
Palivizumab [7]	Solution injection vial	Synagis®	2°C to 8°C	14 days	
Panitumumab	Solution injection vial	Vectibix®	2°C to 8°C	24 hrs	SmPC
Patiromer	Packet, oral	Veltassa®	2°C to 8°C	25°C±2°C up to 3 months	SmPC
Pegaspargase	Solution injection vial	Oncaspar®	2°C to 8°C	Up to 25°C for 48 hours	SmPC
Pegfilgrastim	Prefilled syringe	Neulasta®	2°C to 8°C	Up to 48 hours	SmPC
Peginterferon Alfa-2a [7]	Prefilled syringe	Pegasys®	2°C to 8°C	24 hours	
Pembrolizumab	Solution injection vial	Keytruda®	2°C to 8°C	24 h	SmPC
Pertuzumab, Trastuzmab and Hyaluronidase	Solution injection vial	Phesgo	2 to 8 °C	24 h	SmPC
Pneumococcal Conjugate Vaccine (13-Valent)	Solution injection vial	Prevnar 13™	2 to 8 °C	Up to 25°C: 4 days	SmPC
Pneumococcal Polysaccharide Vaccine (23-Valent) [8]	Solution injection vial	Pneumovax® 23	2°C to 8°C	12 h	
Propofol	Injection emulsion	Diprivan®	4°C to 25°C	4°C to 25°C	SmPC
Prothrombin Complex Concentrate (Human) [(Factors II, VII, IX, X), Protein C, and Protein S]	Solution injection vial	Kcentra®	2°C	25°C	SmPC
Prothrombin Complex Concentrate (Human) [(Factors II, VII, IX, X), Protein C, and Protein S]	Injection vial	Octaplex	2°C	25°C	SmPC
Rabies Immune Globulin (Human)	Solution injection vial	Kedrab®	2°C to 8°C	Up to 25°C: 1 month	SmPC
Rabies Immune Globulin (Human)	Solution injection vial	Hyperrab®	2°C to 8°C	Up to 25°C: 6 month	SmPC
Ranibizumab	Solution intravitreal	Lucentis®	2°C to 8°C	24 h	SmPC
Ranibizumab	Solution intravitreal	Byoovis®	2°C to 8°C	30°C up to 60 days	SmPC
Recombinant Follicle Stimulating Hormone	Prefilled syringe	Gonal-F®	2°C to 8°C	20°C to 25°C	SmPC
Remifentanil	Injection vial	Ultiva®	2°C	25°C	SmPC
Risankizumab	Solution Auto-injector	Skyrizi®	2°C to 8°C	24 h	SmPC
RisperiDONE	Vial kit	Risperdal® Consta®	2°C to 8°C	Up to 7 days	SmPC
Rituximab	Solution injection vial	Rituxan®	2°C to 8°C	24 h	SmPC
Rituximab and hyaloronidase	Solution injection vial	Rituxan Hycela®	2°C to 8°C	8 hours	SmPC
Rocuronium	Solution injection vial	Zemuron®	2°C to 8°C	Up to 60 days/opened vials within 30 days	SmPC
RomiPLOStim	Solution injection vial	Nplate	2°C to 8°C	25°C up to 30 days	SmPC
Romosozumab	Prefilled syringe	Evenity	2°C to 8°C	25°C up to 30 days	SmPC
Secukinumab	Prefilled syringe	Cosentyx®	2°C to 8°C	Up to 4 days	SmPC
Semaglutide	Solution Pen-injector	Ozempic	2°C to 8°C	After first use, 56 days	SmPC
Semaglutide	Solution Pen-injector	Wegovy	2°C to 8°C	28 days	SmPC
Sirolimus	Solution, Oral	Rapamune	2°C to 8°C	Up to 25°C: 15 days	SmPC
Somapacitan	Pen-injector	Sogroya®	2°C to 8°C	Up to 25°C: 72 h (3 days)	SmPC
Somatropin (Synthetic GH)	Solution reconstituted injection	Genotropin®	2°C to 8°C	20°C to 25°C up to 3 months	SmPC
Somatropin (Synthetic GH)	Solution reconstituted, injection	Norditropin®	2°C to 8°C	Up to 25°C: 3 weeks	SmPC
Succinylcholine	Solution injection vial	Anectine®	2°C to 8°C	14 days	SmPC
Teriparatide	Prefilled syringe	Forteo	2°C to 8°C	36 hours, provided the temperature that the teriparatide is exposed to during travel does not exceed 77°F or 25°C	SmPC
Thiotepa	Solution reconstituted, injection	Tepadina®	2°C to 8°C	4 h	SmPC
Tobramycin (Oral Inhalation)	Nebulization solution, inhalation	Tobi®	2°C to 8°C	Up to 25°C: 28 days	SmPC
Tocilizumab	Prefilled syringes and autoinjector	Actemra®	2°C to 8°C	30°C up to 14 days	SmPC
Tocilizumab	Prefilled syringes and autoinjector	Tyenne®	2°C to 8°C	25°C up to 14 days	SmPC
Trastuzumab and Hyaluronidase	Solution injection vial	Herceptin Hylecta™	2°C to 8°C	30°C up to 4 h	SmPC
Ustekinumab [8]	Solution injection vial	Stelara®	2°C to 8°C	8 h	
Ustekinumab	Prefilled Syringe	Stelara®	2°C to 8°C	Stored at ≤30°C, ≤30 days. Product should not be returned to the refrigerator and discarded if not used within the allotted timeframe. Diluted solution for up to 7 hours	SmPC
Vasopressin	Solution injection vial	Vasostrict®	2°C to 8°C	20°C to 25°C up to 12 months	SmPC
Vedolizumab [8]	Prefilled syringe	Entyvio	2°C to 8°C	48 h	
Vinorelbine	Solution injection vial	Navelbine®	2°C to 8°C	Up to 25°C up to 72 hours	SmPC
Yellow Fever Vaccine	Injection, powder for reconstitution	YF-Vax®	2°C to 8°C	14 days at 35°C-37°C / 3 days to 4.5 days at 45°C-47°C.	SmPC

Discussion

This study presents a reference table based on our institution’s formulary, designed to assist healthcare professionals in managing scenarios where medications labeled for refrigerated storage are inadvertently exposed to room temperature. Such excursions may arise due to routine handling errors, power outages, equipment malfunctions, natural disasters, or failure to recognize the requirement for cold storage. According to manufacturers’ guidance, the medications included in the table may be returned to refrigeration within specified timeframes without compromising their chemical stability or labeled shelf life. Although some pharmaceutical products have stability data available after initial use or reconstitution, such information was not included in this review, as the focus of this article is limited to intact, unopened products intended for storage in warehouse or central pharmacy settings.

An analysis of room temperature stability data shows a clear distribution across time categories. Approximately 22.8% of products remained stable for less than one day or a day, while 24.8% showed stability for two to seven days. The largest group, 34.5%, demonstrated stability within the eight- to 30-day range, making this the most common category. Medium-term stability of one to three months was reported in 11.7% of entries, while four to 12 months' stability accounted for 5.5%. Only 0.7% of cases extended beyond one year. This precise quantification highlights that the majority of products (nearly 60%) have stability ranging between one week and one month, underscoring the importance of product-specific data for inventory and cold-chain management decisions. Our objective was to evaluate the room temperature stability of refrigerated medications prior to first use, to support decision-making related to inventory management and cold chain excursions at the distribution level. Strict monitoring and documentation of excursion duration are essential to ensure the product’s stability and therapeutic integrity throughout its shelf life. The findings of this review can also guide practical strategies, including reducing excess stock to minimize wastage, distributing refrigerated storage units across multiple sites to enhance flexibility, and improving redistribution policies to meet demand in high-use areas. Incorporating this evidence into contingency planning supports operational efficiency, cost reduction, and patient safety. Strict monitoring and documentation of excursion duration are essential to ensure the product’s stability and therapeutic integrity throughout its shelf life [13]. It is important to note that some biosimilars were included in this review, and their stability profiles may differ from those of the reference (originator) biologics due to differences in formulation components, excipients, or manufacturing processes. Therefore, caution should be exercised when extrapolating stability data from originator biologics to their biosimilar counterparts, and product-specific data should be consulted whenever possible. It is important to emphasize that brand-specific stability data must be reported individually, as different products containing the same active substance may have significantly different stability profiles. This is clearly illustrated in the case of somatropin (recombinant human growth hormone). Somatropin products exhibit manufacturer-specific stability profiles at room temperature prior to use: Genotropin® GoQuick (Pfizer, New York, USA) may be stored at room temperature for up to one month; Norditropin® FlexPro (Novo Nordisk, Bagsværd, Denmark) must be refrigerated at 2°C to 8°C and does not permit room temperature storage before use; Saizen® (Merck Serono, Geneva, Switzerland) may be stored at room temperature (15°-30°C) with expiry as stated on the label; whereas Omnitrope® (Sandoz, Basel, Switzerland) requires continuous refrigeration (2°C-8°C) and does not allow storage at room temperature prior to reconstitution. These distinctions highlight the necessity of consulting manufacturer-specific data and avoiding generalizations across products, even when they share the same active ingredient [13-16]. Haemocomplettan® was the first fibrinogen concentrate introduced and required refrigerated storage. In contrast, RiaSTAP® (2009) and FIBRYGA® (2017) did not originally require refrigeration, but were mentioned in our analysis for differentiation and completeness. According to the manufacturer, Vyzulta (latanoprostene bunod ophthalmic solution, 0.024%, Bausch + Lomb, Laval, Canada) may be exposed to temperatures up to 40 °C (104 °F) for a period not exceeding 14 days during shipping [17]. This indicates that short-term excursions to elevated temperatures are acceptable under controlled conditions. Following such an excursion, the product may be returned to refrigerated storage (2-8 °C), provided that the cumulative exposure does not exceed the specified limit. However, it should be noted that returning the product to refrigeration does not reset the allowable excursion period, as chemical degradation may be cumulative.

Although certain pharmaceutical products are labeled as stable at room temperature for a defined duration, the permissibility of returning them to refrigerated conditions thereafter remains unclear. The practice of re-refrigeration does not restore the product’s original shelf life, as any degradation occurring during the room temperature exposure may be cumulative and irreversible. Notably, most manufacturers and official product labeling do not provide explicit guidance on the acceptability of re-refrigeration following temperature excursions. In the absence of such clarification, it is scientifically prudent to consider the allowed room temperature exposure as a non-reversible, cumulative limit. Our findings indicate that the majority of thermolabile pharmaceutical products maintain acceptable stability at ambient conditions for at least 24 hours. Furthermore, a substantial proportion of outpatient medications distributed by hospital pharmacy services exhibit robust stability at room temperature over extended durations. These observations suggest that common temperature deviations resulting from patient-related factors or technical failures do not invariably necessitate the disposal of affected products.

Considering the increasing prevalence and high cost of biologics and other temperature-sensitive agents, minimizing unnecessary wastage is of significant clinical and economic importance. While standard storage conditions are well documented in regulatory prescribing information and reference compendia, data on temporary deviations from recommended temperatures are often lacking or inconsistently reported. In many instances, excursion data had to be obtained via direct communication with manufacturers; however, this process was frequently impeded by delayed responses, unavailability of data, or restrictions on information disclosure due to regulatory or legal constraints. Notably, 17% of the refrigerated medications evaluated lacked retrievable excursion stability data, despite direct inquiry. Additionally, reliance on tertiary databases such as Lexicomp and IBM Micromedex posed limitations, as some entries were outdated or incomplete at the time of review. Although prior studies have explored similar themes, they have generally focused on narrower selections of drugs, often limited to those included in local hospital formularies. Our study builds upon these efforts by providing a more extensive and updated compilation of stability data, thereby offering a comprehensive and practical resource for pharmacy practitioners and clinical decision-makers.

Given the exponential rise in the number and cost of refrigerated pharmaceuticals, the need for publicly available stability data under non-standard conditions is increasingly urgent. We advocate for pharmaceutical manufacturers to proactively publish the results of controlled stability studies conducted outside the standard storage conditions listed in the SmPC, even when such data are accompanied by appropriate disclaimers. This would support evidence-based decisions and reduce the incidence of preventable drug loss. Although this review was not conducted using a formal systematic methodology, which may have led to the omission of relevant published data, the limited scope of available literature underscores the value of our findings. In light of this, we recommend that healthcare providers consult manufacturers directly when cold chain breaches occur, particularly in the context of high cost or clinically sensitive therapeutics.

## Conclusions

The reference tool developed through this study provides timely and clinically relevant data to support informed decision-making regarding the continued use of refrigerated medications following temporary storage deviations. Its application in routine practice may contribute significantly to cost containment, medication safety, and operational efficiency within hospital pharmacy settings. Future research should focus on conducting systematic studies under home-use conditions and promoting global harmonization of excursion data to further strengthen evidence-based practice.
